# Unpacking feedback literacy in EFL reading: structural relationships and effective strategies among Chinese college students and English instructors

**DOI:** 10.3389/fpsyg.2026.1821661

**Published:** 2026-05-04

**Authors:** Chunxiang Fan, Jinyan Huang, Jai Kumar, Ying Liu, Qiong Zhu, Xiangting Tang, Yiwa Fan

**Affiliations:** 1 Hunan University, Changsha, China; 2 Jiangsu University, Zhenjiang, China; 3 Xiangnan University, Chenzhou, China; 4 The Chinese University of Hong Kong, Hong Kong, Hong Kong SAR, China; 5 Changde College, Changde, China

**Keywords:** appreciating feedback, Chinese university students, EFL reading, feedback literacy, making judgments, managing affect, necessary condition analysis, PLS-SEM

## Abstract

This mixed-methods study examined feedback literacy in English-as-a-foreign-language (EFL) reading among Chinese non-English-major undergraduates. Drawing on Carless and Boud’s four-dimensional framework, the study investigated the structural relationships among appreciating feedback (AF), making judgments (MJ), managing affect (MA), and taking action (TA), and explored how students and teachers described actionable feedback practices in reading contexts. Survey data were collected from 1,596 students using a 20-item questionnaire adapted to EFL reading, together with two open-ended questions. Semi-structured interviews were also conducted with 20 college English instructors. Quantitative data were analyzed using partial least squares structural equation modeling (PLS-SEM), multigroup analysis, and necessary condition analysis (NCA), while qualitative data were thematically coded. The results showed that AF, MJ, and MA were positively associated with TA, with MA emerging as the most proximal predictor of action-taking. AF also influenced TA indirectly through MJ and MA. Multigroup analysis indicated small but significant differences across gender, year of study, and CET-4 status. NCA further suggested that AF, MJ, and MA functioned as necessary conditions at different points in the feedback process. Qualitative findings showed that students acted on feedback by reviewing comments, setting goals, seeking clarification, using supplementary resources, and reflecting on progress, while instructors promoted action-taking through clear feedback, follow-up tasks, resource provision, and structured reflection. The study extends feedback-literacy research to the underexplored domain of EFL reading and offers context-sensitive implications for supporting feedback uptake in Chinese university classrooms.

## Introduction

Feedback is widely regarded as a central mechanism through which assessment supports learning. In higher education, and particularly in language education, feedback is expected to help students identify strengths and weaknesses, regulate learning, and improve performance over time (Carless and Boud, 2018; Hattie and Timperley, 2007; Shute, 2008). In EFL contexts, feedback can support language development by helping learners refine strategies, notice gaps, and revise performance in relation to instructional goals (Gan, 2020; Han and Xu, 2019; Wu et al., 2021). Research on EFL reading similarly suggests that feedback can improve comprehension, strategy use, and metacognitive monitoring (Cai et al., 2023; Swart et al., 2019, 2022).

However, feedback does not automatically lead to improvement. A recurring finding in higher education is that feedback often fails to generate meaningful change because students do not understand it, do not trust it, do not know how to use it, or do not act on it (Boud and Molloy, 2013a; Carless, 2015; Winstone et al., 2017). This problem is especially salient in EFL settings, where feedback may be complicated by linguistic difficulty, face concerns, power asymmetries, and emotional reactions to correction. Feedback effectiveness therefore depends not only on what teachers provide, but also on what students are able and willing to do with it.

This concern has led researchers to focus increasingly on feedback literacy, usually defined as the understandings, capacities, and dispositions needed to interpret feedback and use it productively (Carless and Boud, 2018; Molloy et al., 2019). The concept shifts attention away from feedback as a one-way transmission and toward the learner’s active role in recognizing value, making judgments, regulating emotion, and taking action. In this sense, feedback literacy is closely related to self-regulation, evaluative judgment, and learner agency.

Despite growing interest in the concept, three gaps remain. First, feedback-literacy research in applied linguistics has focused mainly on writing, especially written corrective feedback, peer review, and automated feedback systems. Reading has received much less attention, even though reading feedback often concerns comprehension, inference, vocabulary, and strategy use rather than visible product errors. Second, much of the literature is either conceptual or based on relatively small-scale qualitative designs, leaving limited evidence on the structural relationships among the major dimensions of feedback literacy. Third, although feedback literacy is often framed as a student capacity, less is known about the practical strategies through which students and teachers jointly translate feedback into concrete reading-related action.

These gaps are especially relevant in the Chinese university EFL context. Chinese non-English-major undergraduates typically experience large-scale College English instruction, high-stakes proficiency pressures, and learning cultures shaped by teacher authority, examination orientation, and concerns about face. Such contextual conditions may influence how students value, interpret, feel about, and act on feedback. They may also shape the feasibility of classroom practices intended to support feedback literacy.

Against this background, the present study examines feedback literacy among Chinese non-English-major college students in EFL reading. It focuses on the four dimensions proposed by Carless and Boud (2018): appreciating feedback (AF), making judgments (MJ), managing affect (MA), and taking action (TA). To address the gaps identified above, the study combines quantitative modeling with qualitative evidence from students and instructors.

## Literature review

To position the study more clearly, this section first reviews key perspectives on feedback, then considers the concept of feedback literacy, and finally examines how the four dimensions may operate in EFL reading.

### Feedback as information, dialogue, and learner process

Definitions of feedback differ across research traditions. In a classic cognitive view, feedback is information about the gap between current and desired performance that can be used to improve future work (Hattie and Timperley, 2007; Narciss, 2013; Shute, 2008). This definition remains influential because it highlights the informational value of feedback and its role in closing learning gaps. At the same time, it can risk reducing feedback to a message delivered by an expert.

A dialogic perspective broadens the concept by treating feedback as an interactive and socially mediated process rather than a one-way transmission (Carless, 2015; Yang and Carless, 2013). From this perspective, effectiveness depends on opportunities for dialogue, clarification, comparison, and uptake. A related learner-centered perspective focuses on what students actually do with feedback. De Kleijn (2023), for example, describes feedback processing as involving gathering, interpreting, using, and responding to feedback information. These perspectives are particularly relevant in EFL learning, where students may receive comments from teachers, peers, or digital tools but still fail to convert them into learning gains.

### Feedback literacy and its conceptual tensions

Carless and Boud (2018) define feedback literacy as the understandings, capacities, and dispositions needed to make sense of feedback and use it to improve work or learning strategies. Their framework identifies four dimensions: appreciating feedback, making judgments, managing affect, and taking action.

The model has become highly influential because it moves beyond teacher delivery and highlights student agency. It has also been extended by later work emphasizing learning-centered feedback environments and ecological perspectives (Chong, 2021; Molloy et al., 2019). Even so, the model has limitations. First, it was proposed primarily as a conceptual framework rather than a strongly specified causal model. Second, the four dimensions are analytically distinct but may overlap in practice, especially when measured through self-report. Third, the framework foregrounds student capacities but can underplay the role of task design, teacher mediation, and institutional culture in enabling or constraining feedback uptake.

A related debate concerns where responsibility for feedback success should lie. One line of research emphasizes student recipience and the need to foster active, agentic engagement (Winstone et al., 2017). Another emphasizes that students can exercise such agency only when teachers create usable, low-ambiguity, and dialogic feedback opportunities (Boud and Molloy, 2013b; Yang and Carless, 2013). The present study adopts the view that student feedback literacy and teacher feedback design are mutually constitutive rather than oppositional.

### Feedback literacy in EFL learning

In EFL research, feedback has been examined extensively in writing, including teacher written feedback, peer feedback, and automated feedback (Cui et al., 2021; Xu et al., 2022). These studies show that feedback can support revision and performance, but that its value depends on learner uptake. Research on feedback engagement in EFL settings also suggest that motivation, self-efficacy, task value, and emotional response shape how students seek, interpret, and use feedback (Gan et al., 2023; Han and Xu, 2019; Papi et al., 2019).

By contrast, feedback literacy in EFL reading remains comparatively underdeveloped. This omission is important because reading feedback often concerns strategy selection, inferencing, text comprehension, and metacognitive monitoring rather than only visible form-based errors. Feedback on reading therefore requires students not only to understand comments but also to re-evaluate comprehension processes, regulate frustration, and adopt new strategies. Meta-analytic evidence suggests that feedback can improve reading comprehension and reading-strategy use, especially when feedback is timely and comprehension-focused (Swart et al., 2019, 2022). Still, we know relatively little about the learner capacities that enable such feedback to be acted upon.

### The four dimensions in EFL reading

#### Appreciating feedback (AF)

Appreciating feedback refers to students’ recognition that feedback is valuable, usable, and worth attending to. In Chinese EFL settings, students often value teacher feedback, especially when it is perceived as credible, specific, and useful for examination performance or skill development (Gan, 2020; Lu et al., 2022). Appreciating feedback is not simply liking feedback. It also involves understanding that feedback may come from different sources, take different forms, and support performance rather than only immediate correction.

#### Making judgments (MJ)

Making judgments refers to evaluative judgment: the ability to assess the quality of one’s own work and that of others. In language education, this includes comparing responses, interpreting criteria, and diagnosing strengths and weaknesses. Peer assessment and self-assessment may strengthen evaluative judgment, but the benefits depend on students’ readiness, trust, and skill (Li et al., 2022; Ritonga et al., 2022). In reading, MJ can involve recognizing whether one has misidentified a main idea, misunderstood a lexical cue, or failed to infer author intention.

#### Managing affect (MA)

Managing affect refers to students’ ability to regulate emotional reactions to feedback, especially when feedback is critical or threatening. This dimension is particularly important in EFL classrooms, where correction may trigger embarrassment, anxiety, or defensiveness. Students who can tolerate uncertainty and criticism may be better positioned to process and use feedback constructively. Conversely, negative affect may block feedback uptake even when students understand feedback cognitively (Han and Xu, 2019; St Clair-Thompson and Devine, 2023).

#### Taking action (TA)

Taking action is the behavioral dimension of feedback literacy. It refers to using feedback to modify strategies, revise work, seek support, or plan future improvement. In reading, TA may include re-reading with a new purpose, annotating texts differently, practicing inference, reviewing vocabulary patterns, or using supplementary resources. Because action is the point at which feedback becomes instructionally consequential, it is arguably the most educationally important dimension.

### Interrelationships among the four dimensions

Although the four dimensions are conceptually distinct, they are likely interdependent. Appreciating feedback may support making judgments because students who see feedback as useful may be more willing to compare, scrutinize, and reflect on work quality. Making judgments may support managing affect because clearer judgment can reduce uncertainty and help students interpret criticism more productively. Managing affect is likely to be especially important for taking action because students may understand feedback intellectually but still fail to act on it if they feel discouraged, ashamed, or overwhelmed.

At the same time, these relationships should not be assumed to be automatic or purely linear. Appreciating feedback may coexist with passivity. Making judgments may expose weaknesses and intensify anxiety. Taking action may depend on contextual support rather than only on individual dispositions. These possibilities justify examining the relationships empirically and interpreting them cautiously.

### Conceptual framework and research questions

Building on the literature above, the present study uses Carless and Boud (2018) framework, informed by self-regulation theory (Zimmerman, 2000), to examine the relationships among AF, MJ, MA, and TA in EFL reading. Rather than claiming definitive causality, the study tests theory-informed directional relationships and complements them with qualitative evidence and necessary condition analysis.

The study addresses three research questions (RQs). RQ1: what structural relationships exist among appreciating feedback (AF), making judgments (MJ), managing affect (MA), and taking action (TA) in EFL reading? RQ2: Do these relationships differ across gender, year of study, and English proficiency (CET-4 status)? And RQ3: What strategies do Chinese non-English-major college students use to act on feedback in EFL reading, and how do English instructors encourage such action-taking?

### Hypotheses

Based on the literature reviewed above, the following hypotheses were examined:

*H1*: AF is positively associated with MJ.

*H2*: AF is positively associated with MA.

*H3*: MJ is positively associated with MA.

*H4*: MA is positively associated with TA.

*H5*: AF influences TA indirectly through MA.

*H6*: AF influences MA indirectly through MJ.

*H7*: MJ influences TA indirectly through MA.

*H8*: AF influences TA indirectly through the sequential path MJ → MA.

These hypotheses are grounded in self-regulation and evaluative-judgment theory, but they were tested with the recognition that the strength of these relations may vary by context and student subgroup.

## Methodology

### Research ethics

This study involving human participants was reviewed and approved by the Research Ethical Review Board of the corresponding author’s research organization. Participants were informed about the purpose of the study, the voluntary nature of participation, and the confidentiality of their responses.

### Participants

A total of 2,105 Chinese non-English-major undergraduates responded to the online questionnaire. After 507 responses were removed because they displayed identical answers across items or otherwise suggested insufficient response quality, 1,596 cases were retained for analysis. Of these, 668 were male (41.8%) and 922 were female (58.2%). In terms of year of study, 634 were freshmen (40.2%) and 960 were sophomores (59.8%). Regarding English proficiency, 991 students (62.0%) had passed CET-4 and 605 (38.0%) had not.

Participants were recruited through university-affiliated WeChat groups with assistance from professors and advisers in different parts of China. Because recruitment relied on convenience-based online dissemination rather than institutional sampling, the sample should be viewed as large and geographically heterogeneous but non-probabilistic. The design therefore supports analytic insight into the structure of feedback literacy, but not strict population-level representativeness.

In addition, 20 college English instructors from 20 universities took part in semi-structured interviews. Thirteen were female and seven were male. Sixteen held graduate degrees and four held undergraduate degrees in EFL-related fields. Thirteen had more than 10 years of experience teaching college EFL reading.

### Instrument

The questionnaire included 20 five-point Likert items (1 = strongly disagree to 5 = strongly agree) designed to measure AF, MJ, MA, and TA in the specific context of English reading. The items were adapted from Carless and Boud (2018) feedback-literacy framework by rewriting them to refer explicitly to English reading tasks, reading homework, reading-related feedback, and action-taking in reading (see Appendix A).

Because the original framework was not designed specifically for EFL reading, this adaptation should be understood as a contextualization rather than full independent scale-development. The instrument therefore provides useful evidence for the present sample, but future studies should continue to test its cross-context validity, especially in non-Western EFL settings and through additional forms of validation beyond internal-consistency statistics.

The survey also included two open-ended questions asking students how they usually act on feedback from teachers and peers to improve their English reading. The instructor interviews focused on strategies used to encourage students to act on feedback.

### Data collection

The student questionnaire was distributed through Survey Star, an online platform, and circulated through WeChat groups associated with university students. Participation was voluntary and anonymous. The interview data from instructors were collected through semi-structured interviews focused on classroom practices that promote feedback uptake in EFL reading.

### Data analysis

This study adopted a mixed-methods design combining partial least squares structural equation modeling (PLS-SEM), multigroup analysis, necessary condition analysis (NCA), and qualitative thematic analysis. PLS-SEM was selected because the study aimed to test a theory-informed but still developing structural model and because the model included multiple latent constructs and mediation paths (Hair et al., 2022). It was used to estimate the structural relations among AF, MJ, MA, and TA and to test indirect effects. Because the data were cross-sectional, the results are interpreted as directional structural associations consistent with theory rather than as strong causal claims.

NCA was used to complement PLS-SEM. Whereas PLS-SEM estimates whether one construct is sufficient to predict variance in another, NCA examines whether a minimum level of one condition is required for a given outcome to be attainable (Dul, 2022; Richter and Hauff, 2022; Richter et al., 2020). In the present study, NCA was used to identify bottlenecks in the feedback-literacy process.

All quantitative analyses were conducted in SmartPLS 4.0 (Ringle et al., 2022). The researchers first assessed the measurement model and then estimated the structural model. Multigroup analysis examined whether path estimates differed across gender, year of study, and CET-4 status.

The qualitative data from open-ended responses and instructor interviews were coded thematically. Responses were read repeatedly, categorized into recurring themes, and compared across coders before final thematic consolidation. The qualitative findings were then used to help explain and contextualize the quantitative results (Creswell and Creswell, 2023).

### Generalizability and methodological cautions

Three methodological cautions should be noted at the outset. First, the sampling approach relied on voluntary participation through WeChat-based convenience recruitment, which limits external validity. Second, the study relied primarily on self-report data, which may not fully capture actual feedback behavior. Third, because all focal student constructs were measured using the same instrument at one time point, common method bias cannot be ruled out completely. These issues are revisited in the limitations section.

## Results

To improve readability, the results section reports only the most salient findings in the text and leaves the full numerical detail to the tables.

### Measurement model

Reliability and convergent validity were examined through item loadings, Cronbach’s alpha, composite reliability, and average variance extracted (AVE). As shown in Table 1, Cronbach’s alpha values ranged from 0.787 to 0.823 and composite reliability values ranged from 0.853 to 0.876, indicating satisfactory internal consistency (Hair, 2019).

**Table 1:** Reliability and convergent validity of the four feedback-literacy constructs.

Constructs	Items	Loading	Cronbach’s alpha	Composite reliability	Average variance extracted (AVE)
AF	AF1	0.737	0.804	0.865	0.561
	AF2	0.768			
	AF3	0.719			
	AF4	0.744			
	AF5	0.776			
MA	MA1	0.753	0.793	0.853	0.494
	MA2	0.742			
	MA3	0.751			
	MA4	0.741			
	MA5	0.63			
	MA6	0.581			
MJ	MJ1	0.779	0.787	0.862	0.611
	MJ2	0.801			
	MJ3	0.772			
	MJ4	0.773			
TA	TA1	0.744	0.823	0.876	0.587
	TA2	0.797			
	TA3	0.776			
	TA4	0.793			
	TA5	0.716			

AF, appreciating feedback; MJ, making judgments; MA, managing affect; TA, taking action.

AVE values were adequate for AF (0.561), MJ (0.611), and TA (0.587). For MA, AVE was 0.494, slightly below the commonly recommended 0.50 threshold. Convergent validity for MA should therefore be regarded as marginal rather than fully satisfactory. The construct was retained, however, because its composite reliability was acceptable and the items captured theoretically important aspects of affective regulation in feedback settings.

Discriminant validity was assessed using the Fornell–Larcker (1981) criterion. As shown in Table 2, the square roots of AVE were higher than the corresponding inter-construct correlations, supporting acceptable discriminant validity.

**Table 2:** Discriminant validity of the feedback-literacy constructs based on the Fornell–Larcker criterion.

Constructs	AF	MA	MJ	TA
AF	0.749			
MA	0.773	0.703		
MJ	0.726	0.802	0.781	
TA	0.785	0.805	0.76	0.766

Diagonals (italic) values are the square root of the AVE values of each respective construct.

AF, appreciating feedback; MJ, making judgments; MA, managing affect; TA, taking action.

### Findings for RQ1: structural relationships among AF, MJ, MA, and TA

**Figure 1:**
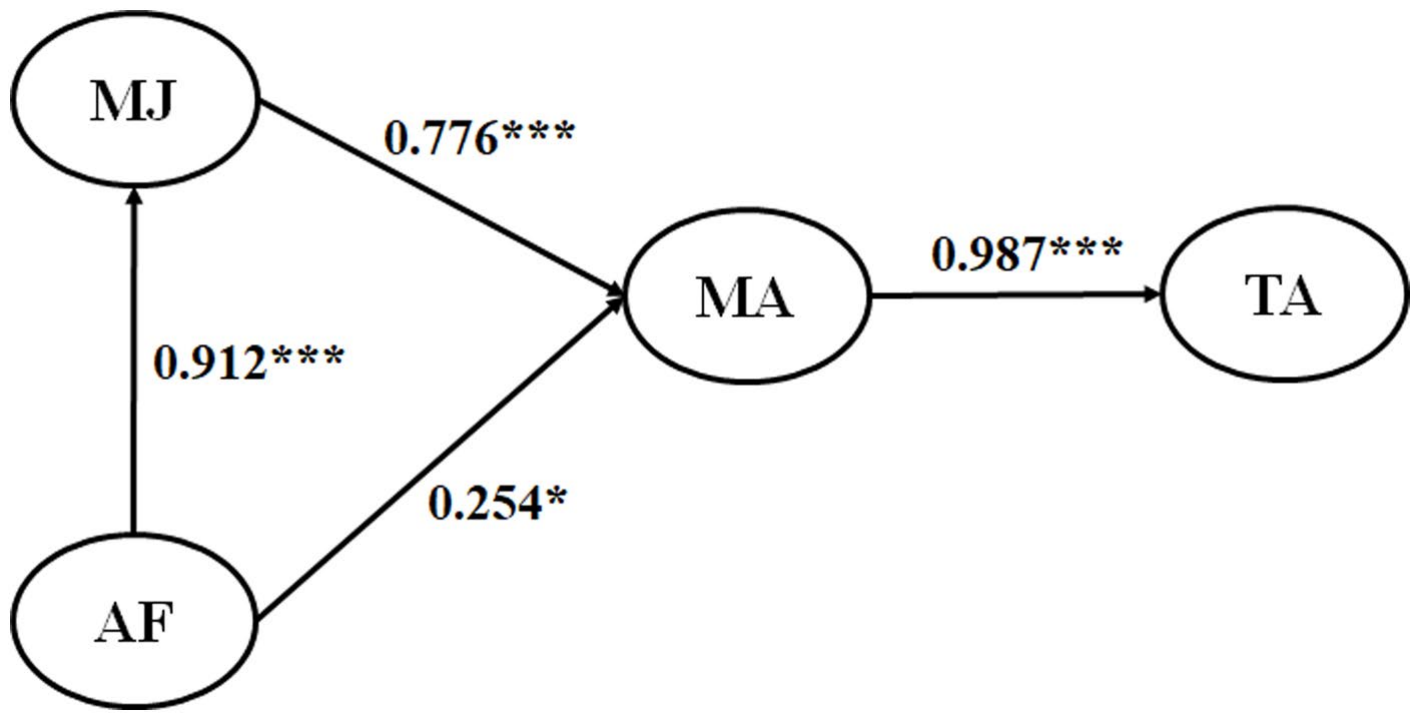
The structural model. AF, appreciating feedback; MJ, making judgments; MA, managing affect; TA, taking action.

The structural model results are reported in Table 3 and summarized visually in Figure 1. All four direct hypotheses were supported. AF was strongly and positively associated with MJ (*β* = 0.912, *p* < 0.01), and it was also positively associated with MA (*β* = 0.254, *p* < 0.05). MJ was positively associated with MA (*β* = 0.776, *p* < 0.01), and MA showed a very strong positive association with TA (*β* = 0.987, *p* < 0.01).

**Table 3:** Direct structural paths and hypothesis-testing results.

Hypothesis	Path	Path coefficients	*T* statistics	*p*-values	Decision
H1	AF → MJ	0.912	68.321	**	Supported
H2	AF → MA	0.254	2.524	*	Supported
H3	MJ → MA	0.776	7.976	**	Supported
H4	MA → TA	0.987	94.375	**	Supported

*p < 0.05; **p < 0.01.

AF, appreciating feedback; MJ, making judgments; MA, managing affect; TA, taking action.

Taken together, these findings suggest that students’ recognition of feedback value is closely tied to evaluative engagement, while managing affect is the most immediate factor associated with converting feedback into action.

#### Mediation analysis

Indirect effects are shown in Table 4. AF influenced TA through MA (*β* = 0.251, *p* < 0.05), AF influenced MA through MJ (*β* = 0.708, *p* < 0.01), MJ influenced TA through MA (*β* = 0.766, *p* < 0.01), and AF influenced TA through the sequential path MJ → MA (*β* = 0.699, *p* < 0.01).

**Table 4:** Indirect effects and bias-corrected confidence intervals.

Paths	Beta	*T* statistics	*p*-values	Bias-corrected CI (95%)	
				Lower	Upper
AF → MA → TA	0.251	2.522	*	0.024	0.413
AF → MJ → MA	0.708	7.432	**	0.555	0.926
MJ → MA → TA	0.766	7.937	**	0.607	0.982
AF → MJ → MA → TA	0.699	7.392	**	0.547	0.916

*p < 0.05; **p < 0.01.

AF, appreciating feedback; MJ, making judgments; MA, managing affect; TA, taking action.

These findings suggest that action-taking is not simply a direct extension of positive attitudes toward feedback. Rather, students appear to move toward action through linked processes involving evaluative judgment and affect regulation.

### Findings for RQ2: multigroup differences

PLS-MGA was used to examine differences in structural paths by gender, years of study, and CET-4 status. Significant differences were found across all three grouping variables, although most were small and should be interested cautiously.

#### Gender

As shown in Table 5, the MA → TA path differed significantly across gender (*β* = 0.044, *p* < 0.05), with female students showing a slightly stronger association between affect management and action-taking. No other path differences reached significance.

**Table 5:** Multigroup analysis by gender.

Path	Path coefficient difference (female–male)	*p*-value
AF → MA	−0.229	n.s.
AF → MJ	0.002	n.s.
**MA → TA**	**0.044**	*
MJ → MA	0.220	n.s.
AF → MA → TA	−0.215	n.s.
AF → MJ → MA	0.201	n.s.
MJ → MA → TA	0.251	n.s.
AF → MJ → MA → TA	0.230	n.s.

*p < 0.05; n.s. = not significant.

AF, appreciating feedback; MJ, making judgments; MA, managing affect; TA, taking action.

The bold values indicate significance.

#### Year of study

Freshmen and sophomores differed on several paths (Table 6). The AF → MA path was stronger among freshmen (*β* = 0.679, *p* < 0.01), whereas the MJ → MA path was stronger among sophomores (*β* = −0.646, *p* < 0.01). Several indirect paths also differed significantly. These results suggest that the feedback process may not be developmentally uniform: students earlier in university may rely more on valuing feedback, whereas more experienced students may rely more on evaluative judgment to regulate affect.

**Table 6:** Multigroup model analysis for years of study.

Path	Path coefficient difference (freshmen–sophomore)	*p*-value
**AF → MA**	**0.679**	**
AF → MJ	−0.018	n.s.
MA → TA	−0.023	n.s.
**MJ → MA**	**−0.646**	**
**AF → MA → TA**	**0.664**	**
**AF → MJ → MA**	**−0.609**	**
**MJ → MA → TA**	**−0.655**	**
**AF → MJ → MA → TA**	**−0.617**	**

*p < 0.05; **p < 0.01; n.s., not significant.

AF, appreciating feedback; MJ, making judgments; MA, managing affect; TA, taking action.

The bold values indicate significance.

#### CET-4 status

As shown in Table 7, only the AF → MJ path differed significantly by CET-4 status (*β* = 0.056, *p* < 0.05), suggesting a small proficiency-related difference in the extent to which valuing feedback translates into evaluative judgment. No other path differences were significant.

**Table 7:** Multigroup analysis by CET-4 passing status.

Path	Path coefficient difference (FAIL–PASS)	*p*-value
AF → MA	0.123	n.s.
**AF → MJ**	**0.056**	*
MA → TA	0.007	n.s.
MJ → MA	−0.112	n.s.
AF → MA → TA	0.123	n.s.
AF → MJ → MA	−0.062	n.s.
MJ → MA → TA	−0.106	n.s.
AF → MJ → MA → TA	−0.056	n.s.

*p < 0.05; n.s., not significant.

AF, appreciating feedback; MJ, making judgments; MA, managing affect; TA, taking action.

The bold values indicate significance.

### Necessary condition analysis (NCA)

**Figure 2:**
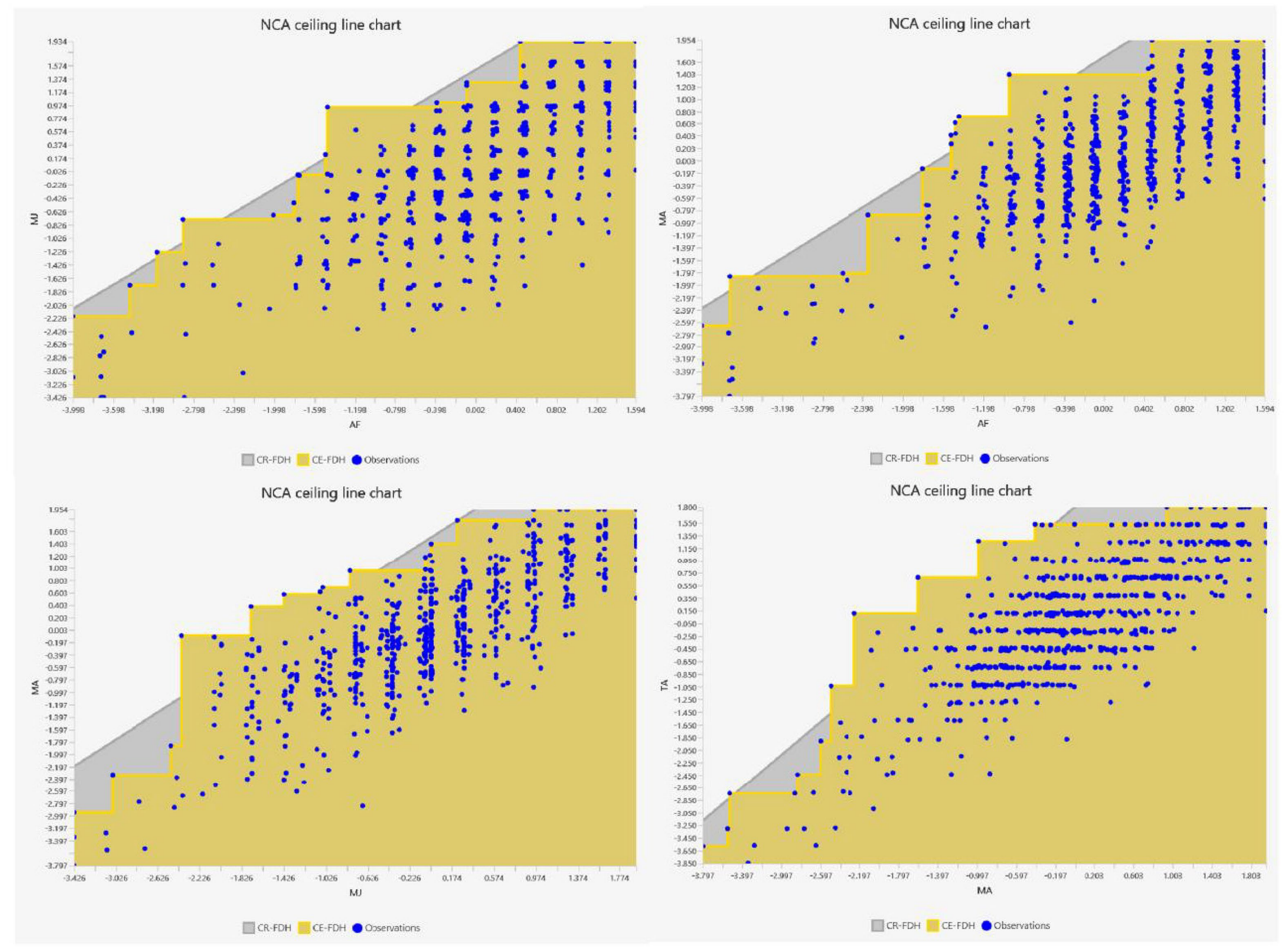
NCA scatterplots and ceiling lines illustrating the necessary-condition relationships among the feedback-literacy dimensions.

NCA was used to examine whether minimum levels of one construct were required for another to reach a given level. As shown in Table 8, AF was a necessary condition for both MJ (d = 0.314, *p* < 0.001) and MA (d = 0.315, *p* < 0.001), while MJ (d = 0.268, *p* < 0.001) and MA (d = 0.281, *p* < 0.001) were necessary conditions for TA. Effect sizes ranged from 0.268 to 0.315 and were all significant. These relationships are visually represented in Figure 2, with the scatter plots and ceiling lines confirming the necessity of these constructs (Dul, 2020).

**Table 8:** NCA effect sizes showing necessary-condition relationships among the feedback-literacy constructs.

Constructs	MJ		MA		TA	
	Effect size	*p* value	Effect size	*p* value	Effect size	*p* value
AF	0.314	0.000	0.315	0.000		
MJ			0.268	0.000		
MA					0.281	0.000

AF, appreciating feedback; MJ, making judgments; MA, managing affect; TA, taking action.

#### Bottleneck analysis

The bottleneck analysis in Table 9 indicates that increasingly high levels of TA require increasingly high levels of MJ and MA, with MA appearing particularly restrictive at higher performance levels. For example, achieving a 90% level of MJ requires over 62% of AF, and achieving peak TA (100%) requires over 80% of both MJ and MA. These results suggest that managing affect is the most constraining condition when very high levels of action-taking are expected.

**Table 9:** Bottleneck levels indicating the minimum percentages of antecedent conditions required for higher outcome levels.

*Y* (%)	MJ	MA		TA
	AF	AF	MJ	MA
0.00%	0	0	0	0
10.00%	0	0	0	0.376
20.00%	0	0.752	0.815	1.065
30.00%	1.19	0.752	1.253	1.566
40.00%	1.441	3.07	1.629	1.692
50.00%	3.509	3.07	1.629	3.258
60.00%	4.135	3.634	1.629	3.258
70.00%	6.767	4.887	4.511	3.258
80.00%	6.767	12.155	16.792	5.514
90.00%	62.281	12.155	46.93	12.531
100.00%	62.281	69.799	80.702	81.83

AF, appreciating feedback; MJ, making judgments; MA, managing affect; TA, taking action.

To summarize the quantitative pattern more clearly, Table 10 juxtaposes the sufficiency logic of PLS-SEM and the necessity logic of NCA.

**Table 10:** Comparison of PLS-SEM and NCA findings for the main construct relationships.

Constructs	Results of the PLS-SEM	Results of the NCA
AF → MJ	Significant	Significant as necessary condition
AF → MA	Significant	Significant as necessary condition
MJ → MA	Significant	Significant as necessary condition
MA → TA	Significant	Significant as necessary condition

AF, appreciating feedback; MJ, making judgments; MA, managing affect; TA, taking action.

### Findings for RQ3: students’ action-taking strategies and instructors’ strategies for promoting action-taking

The qualitative results complement the quantitative findings by showing how action-taking is enacted in practice.

#### Students’ action-taking strategies

Five recurring strategies emerged from students’ open-ended responses.

1. Active engagement with feedback. Many students reported rereading comments carefully and incorporating them into later reading tasks. One sophomore explained that “…*my English teacher emphasized the significance of attending to context clues while reading. I actively honed this skill by annotating unfamiliar words and phrases, deducing their meanings from the surrounding text*.”
2. Goal setting. Approximately half of the students described converting feedback into specific reading goals, such as learning a fixed number of academic words each week or focusing on one comprehension weakness at a time. For example, one freshman student articulated, “…*once my peer suggested that I focus on enhancing my comprehension of academic vocabulary, I promptly set a goal to learn five new academic words each week and integrated them into my reading practice*.”
3. Seeking clarification. A substantial group said they sought further explanation when they did not understand feedback. This helped them move from vague awareness of weakness to clearer action. For example, another freshman student noted, “*My English teacher advised me to improve my inference skills, so I requested specific examples of how to make inferences while reading an English passage*.”
4. Using supplementary resources. Some students expressed that they utilized supplementary resources to reinforce the feedback they received. This could involve using online learning platforms, reference books, or supplementary materials recommended by their teachers or peers. For instance, another sophomore student remarked, “…*when my English teacher suggested that I enhance my understanding of grammar rules, I turned to online grammar exercises and grammar textbooks to practice and reinforce my knowledge*.”
5. Reflecting on progress. A small but meaningful group described tracking progress through reflective journals or reading notes. These practices appear especially relevant to the MA → TA pathway because they may help students persist after receiving critical feedback. For example, a third sophomore student shared, “*I maintained a reflective journal where I tracked my reading progress, recorded the feedback received, and reflected on how I had incorporated suggested improvements into my reading practice*.”

Instructors’ strategies for promoting action-taking.

Five themes also emerged from the instructor interviews.

1. Providing clear and constructive feedback. All instructors emphasized specificity. Rather than vague criticism, they reported giving usable comments linked to strategies or examples. For example, one senior instructor stated, “…*instead of simply noting that a student needs to work on vocabulary, I typically provide feedback such as,* ‘*Your understanding of vocabulary is lacking. Try to focus on context clues to infer the meanings of unfamiliar words.*’”
2. Encouraging active engagement. Many instructors described follow-up activities requiring students to apply feedback, such as revisiting reading passages or discussing comments in pairs. For instance, one young instructor shared, “*After providing feedback on a reading assignment, I typically assign follow-up activities that require students to apply the suggested improvements.*”
3. Setting goal-oriented tasks. Teachers often translated broad feedback into manageable tasks, such as summarizing paragraphs, identifying main ideas, or tracking inference errors over time. For example, another senior instructor stated, “…*if a student struggles with identifying main ideas in a text, I might assign a task where the student summarizes paragraphs or sections and tracks their progress over time*.”
4. Providing access to resources. Several instructors reported recommending websites, practice exercises, and supplementary reading materials to support uptake. For instance, another young instructor explained, “*I typically compile a list of recommended websites or apps for practicing reading comprehension skills and share it with my class*.”
5. Facilitating reflection and self-assessment. Some instructors encouraged students to reflect on how they had used feedback and what remained difficult through through self-assessment activities, reflective journaling, or class discussion. For example, a third senior instructor shared, “…*at the end of a reading unit, I typically ask my students to write a reflection on how they have applied feedback to improve their reading comprehension skills and what they plan to do differently in the future*.”

### Integration of quantitative and qualitative findings

The qualitative findings help illuminate the structural model. Students’ reports of clarification-seeking, goal setting, and reflective monitoring provide concrete examples of TA. Teachers’ emphasis on explicit, low-ambiguity feedback likely supports AF and MJ by making feedback easier to value and interpret. Reflection logs, staged tasks, and supportive debriefing appear to help students manage emotional reactions, thereby supporting the strong MA → TA relation found in the quantitative model. These patterns suggest possible classroom mechanisms through which the four dimensions become linked in practice.

## Discussion and conclusions

The results reported above point to a coherent picture of feedback literacy in EFL reading. This section interprets that picture in relation to previous literature, subgroup variation, and classroom practice.

### Summary of main findings

This study examined feedback literacy in EFL reading among Chinese non-English-major undergraduates by testing the structural relationships among AF, MJ, MA, and TA and by identifying strategies through which students and teachers support feedback uptake. Three main findings stand out.

First, the four dimensions formed an interconnected structure in which AF, MJ, and MA were all positively related to TA. Second, MA emerged as the most proximal factor associated with action-taking, both in the structural model and in the NCA bottleneck results. Third, the qualitative findings showed that action-taking was not abstract but enacted through identifiable classroom and learner practices such as reviewing comments, setting goals, seeking clarification, and reflecting on progress.

### What is new in relation to previous literature

The study contributes to the literature in three ways. First, it extends feedback-literacy research from the heavily studied domain of EFL writing to the relatively underexplored domain of EFL reading. This matters because reading feedback often concerns less visible cognitive processes such as inferencing, monitoring, and strategy use.

Second, the study moves beyond descriptive accounts by testing a structural model of the four dimensions and by combing sufficiency logic (PLS-SEM) with necessity logic (NCA). This makes it possible not only to identify positive associations but also to ask whether certain capacities function as prerequisites.

Third, the study integrates student and teacher accounts to show how feedback literacy is enacted in practice. In doing so, it helps bridge a gap between conceptual discussions of feedback literacy and the concrete classroom routines through which feedback becomes usable.

### Interpreting the central role of affect management

Perhaps the most notable result is the strong relation between MA and TA. This suggests that in EFL reading, the ability to regulate emotional responses to feedback may be the immediate gateway to action. Educationally, this means that students may fail to act on feedback not because they do not understand it, but because they feel threatened, discouraged, embarrassed, or overwhelmed by it.

This finding aligns with self-regulation theory and with previous feedback research highlighting the importance of emotional response in feedback uptake (Han and Xu, 2019; St Clair-Thompson and Devine, 2023). It also helps explain why apparently clear feedback sometimes has limited practical effects. In reading, where comprehension failures may be less visible than writing errors and where students may already feel uncertain about performance, affective regulation may be especially important.

At the same time, alternative explanations should be considered. Because MA was measured through self-report, some of its apparent strength may reflect overlap with broader self-regulatory dispositions. In addition, the marginal AVE for MA suggests that this construct should be interpreted cautiously and re-examined in future research.

### Appreciating feedback and making judgments

The strong AF → MJ relation suggests that students who recognize feedback as valuable are more likely to engage in evaluative judgment. In the Chinese university context, one possible explanation is that feedback appreciation is shaped by longstanding educational norms in which teacher comments are seen as legitimate and consequential. Students who value such comments may therefore be more prepared to compare, evaluate, and diagnose work quality.

However, this relation should not be taken to mean that appreciation automatically yields critique. In some contexts, high appreciation may coexist with passivity or dependence on teacher authority. What appears to matter here is that appreciation is linked to evaluative engagement, not merely to compliance.

### Group differences

The multigroup findings indicate that the structure of feedback literacy is not fully identical across subgroups. Female students showed a slightly stronger MA → TA path, which may reflect differences in self-regulation or willingness to translate reflection into action, although the effect size was modest. Freshmen and sophomores differed on several paths, suggesting that feedback literacy may develop with experience. The stronger AF → MA link among freshmen may indicate greater dependence on the perceived value of feedback in early university study, whereas the stronger MJ → MA link among sophomores may indicate growing reliance on evaluative judgment. The CET-4 finding suggests that proficiency may influence how valuing feedback translates into evaluative capacity.

These subgroup results are informative, but they should be interpreted carefully. The present design does not permit strong developmental or psychological explanations, and the observed differences may also reflect unmeasured contextual factors such as course type, instructor practices, or prior feedback experiences.

### The teacher’s role in student feedback literacy

The qualitative findings emphasize that feedback literacy should not be framed as a student-only responsibility. Students reported acting on feedback more effectively when teachers provided specific comments, follow-up tasks, opportunities for clarification, and resources for improvement. Instructors, in turn, described deliberately designing conditions under which feedback could be used. This reinforces dialogic and ecological perspectives on feedback: student agency matters, but it is cultivated through teacher design and classroom norms rather than assumed in advance.

### Implications for practice

#### For EFL learners

Students can be taught to approach feedback not only as information but as something to work with. Based on the present findings, especially the importance of MA and TA, students may benefit from explicit routines such as:

1. Writing a short feedback-action note after each reading task: What feedback did I receive? What will I do next?
2. Keeping a reading improvement log that tracks recurring comprehension problems and the strategies used to address them;
3. Using a clarification protocol, such as asking one teacher question and one peer question when feedback is unclear’
4. Setting one or two micro-goals after each feedback episode, rather than trying to improve everything at once.

These practices are simple, but they can help students move from receiving comments to regulating emotion and taking action.

#### For EFL educators

To make the practical implications more concrete and realistic, the following classroom practices are proposed.

1. Build AF through low-cost framing routines. At the start of a reading unit, teachers can spend a few minutes explaining what kind of feedback students will receive and how it can support future reading rather than only the current task. Short exemplar-based discussions can show what useful uptake looks like.
2. Support MJ with structured comparison tasks. Instead of asking students to “reflect” in a vague way, teachers can provide a checklist for comparing two reading responses or two summaries. This can develop evaluative judgment without requiring exntensive individual marking.
3. Support MA through emotionally safe feedback routines. Because MA was the strongest predictor of TA, teachers should reduce the threat value of feedback. This does not require therapeutic intervention. It can be done through short written scripts such as: “This comment identifies a growth point, not failure,” or by asking students to revise one manageable aspect first. In contexts shaped by face concerns, private written reflection may be more appropriate than public emotional disclosure.
4. Translate feedback into action using follow-up micro-tasks. Rather than giving comments alone, teachers can attach a short action task, such as re-reading a paragraph and identifying inference cues, summarizing the main idea in one sentence, or annotating where confusion occurred. This is particularly useful in large classes because the same task can be assigned to multiple students.
5. Use tiered rather than fully individualized feedback. Given the heavy workload of Chinese university lecturers, complete personalization is unrealistic. A more feasible approach is to use tiered feedback templates: one set of follow-up activities for vocabulary-related issues, another for inferencing, and another for main-idea recognition.
6. Combine teacher feedback with peer and self-regulation tools. Clarification sheets, peer discussion prompts, and reflection logs can distribute responsibility without removing teacher guidance.

### In the Chinese university context

The Chinese higher-education context often combines large classes, examination pressures, teacher authority, and norms discouraging overt emotional display. Recommendations must therefore be realistic. The present findings do not imply that teachers should provide extensive individualized counseling. Rather, they suggest that even within a more uniform instructional culture, teachers can make feedback more actionable by reducing ambiguity, normalizing difficulty, providing low-burden follow-up tasks, using structured peer discussion, and encouraging written self-monitoring. These are practical ways to support AF, MJ, MA, and TA without greatly increasing workload.

### Limitations

Several limitations should be acknowledged. First, the study relied mainly on self-report data, which capture students’ perceptions and reported tendencies rather than direct observation of how they actually use feedback in reading tasks. Students may therefore overreport constructive behaviors or underreport negative emotional reactions.

Second, the study used a cross-sectional design. Although the structural model was theory-informed, the results should not be interpreted as definitive causal effects. The findings indicate directional associations consistent with feedback-literacy theory, but longitudinal or intervention-based studies are needed to establish temporal ordering more convincingly.

Third, the main student variables were collected through the same instrument and source, so common method bias cannot be ruled out. This means that some observed relations may be inflated by shared method variance.

Fourth, the sample was recruited through WeChat-based convenience sampling. Although large and diverse, it was not nationally representative, and institution-level information was not available for stratified analysis. Generalizability should therefore be approached cautiously.

Fifth, the instrument was adapted from a general feedback-literacy framework to the specific context of EFL reading. While reliability was acceptable overall, the MA construct showed only marginal convergent validity. Future studies should refine and further validate the scale, especially for affective items.

Finally, the Chinese cultural context likely shaped the findings. Respect for teacher authority, concern for face, examination orientation, and norms of emotional restraint may influence both feedback interpretation and feedback-related action. The present results should therefore not be generalized uncritically to other cultural or institutional settings.

## Conclusion

This study examined feedback literacy in EFL reading among Chinese non-English-major college students by testing the structural relationships among appreciating feedback, making judgments, managing affect, and taking action, and by identifying strategies used by students and teachers to promote feedback uptake.

Three conclusions can be drawn. First, feedback literacy in EFL reading is best understood as an interconnected process rather than a set of isolated traits. Second, managing affect appears to play a central role in helping students convert feedback into action. Third, feedback uptake is supported not only by student dispositions but also by teacher practices that make feedback clearer, safer, and easier to act on.

Overall, the study extends feedback-literacy research to an underexplored domain, demonstrates the value of combining PLS-SEM and NCA, and offers practical guidance for supporting feedback use in Chinese university EFL reading classrooms.

## Data Availability

The raw data supporting the conclusions of this article will be made available by the authors, without undue reservation.

## Appendix A

**Table A1:** The instrument.

Item	Description
AF1	I understand and value the role of feedback in improving my English reading.
AF2	I know that feedback about my English reading can come from different sources.
AF3	I use digital tools to access, store, and revisit feedback on my English reading.
AF4	I know that feedback about my English reading can come in different forms.
AF5	I understand and value the role of feedback in becoming an active English reader.
MJ1	I develop the ability to make sound judgments about my peers’ English reading homework.
MJ2	I develop the ability to make sound judgments about my own English reading homework.
MJ3	I participate actively in peer-feedback activities in English reading.
MJ4	I develop evaluative capacity in English reading so that I can make more robust judgments.
MA1	I proactively ask teachers for suggestions about my English reading when needed.
MA2	I develop habits of continuous improvement based on self-feedback in English reading.
MA3	I develop habits of continuous improvement based on teacher and peer feedback in English reading.
MA4	I proactively ask peers for suggestions about my English reading when needed.
MA5	I remain emotionally stable when receiving negative feedback on my English reading.
MA6	I avoid becoming defensive when receiving negative feedback on my English reading.
TA1	I develop strategies for acting on feedback in my English reading.
TA2	I make full use of feedback about my English reading to become a more active reader.
TA3	I draw inferences from feedback about my English reading to improve continuously.
TA4	I use feedback to self-regulate my English reading.
TA5	I recognize the importance of taking action in response to feedback about my English reading.

AF, appreciating feedback; MJ, making judgments; MA, managing affect; TA, taking action.
